# Supplementary figures and images for: Itaconate stabilizes CPT1a to enhance lipid utilization during inflammation
Source: eLife. 2024 Feb 2;12:RP92420. doi: 10.7554/eLife.92420 (PMC10945551; doi:10.7554/eLife.92420)

Figure 2C

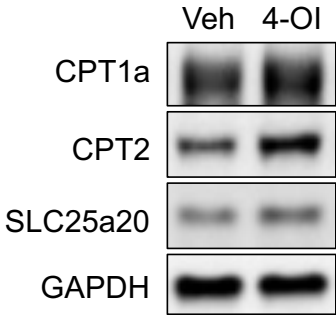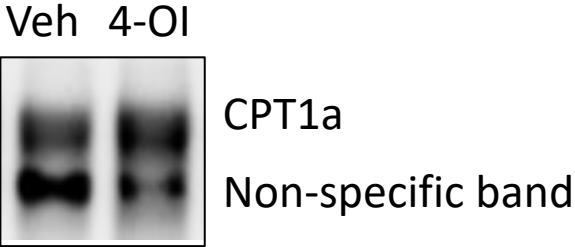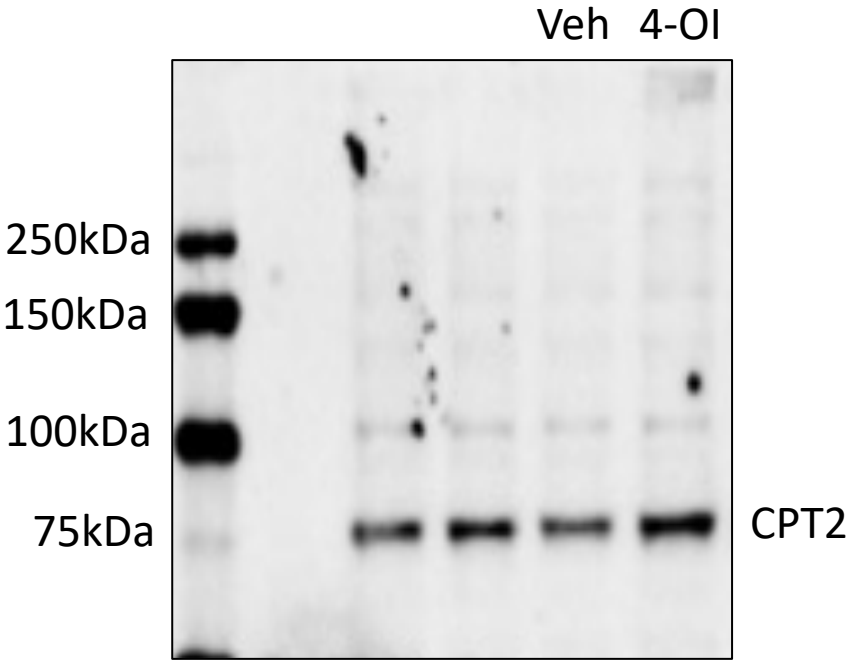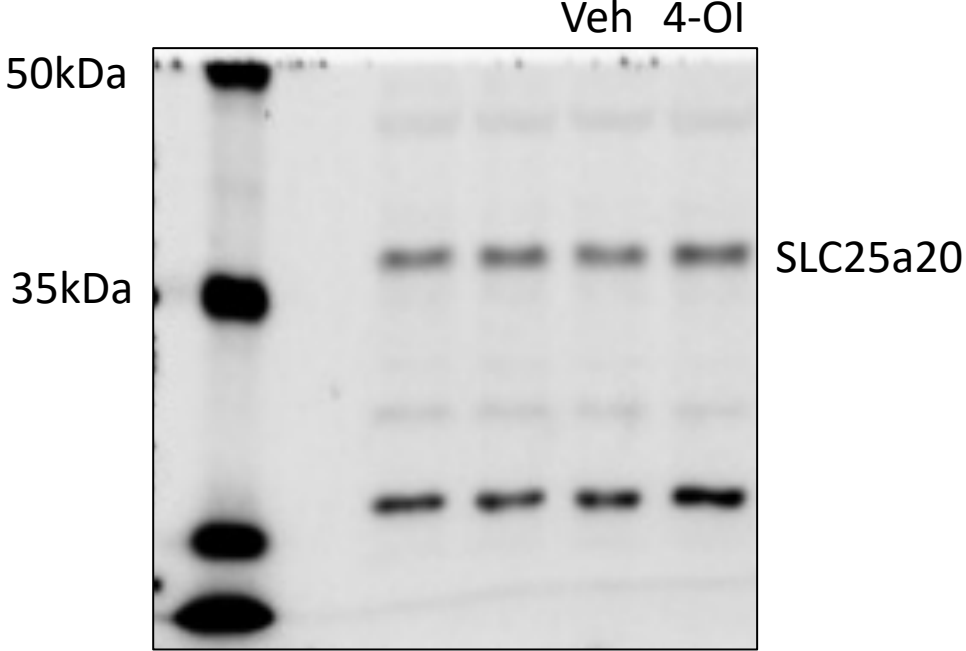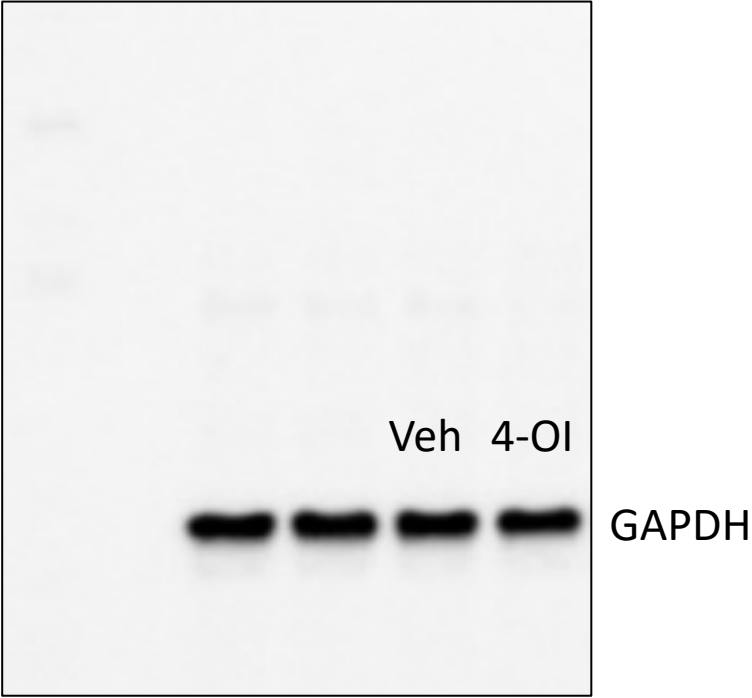

Supplement: Figure 2—source data 1. [file elife-92420-fig2-data1.pdf]

Figure 2D

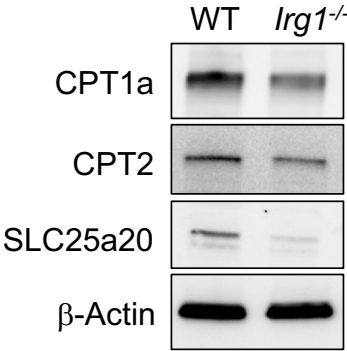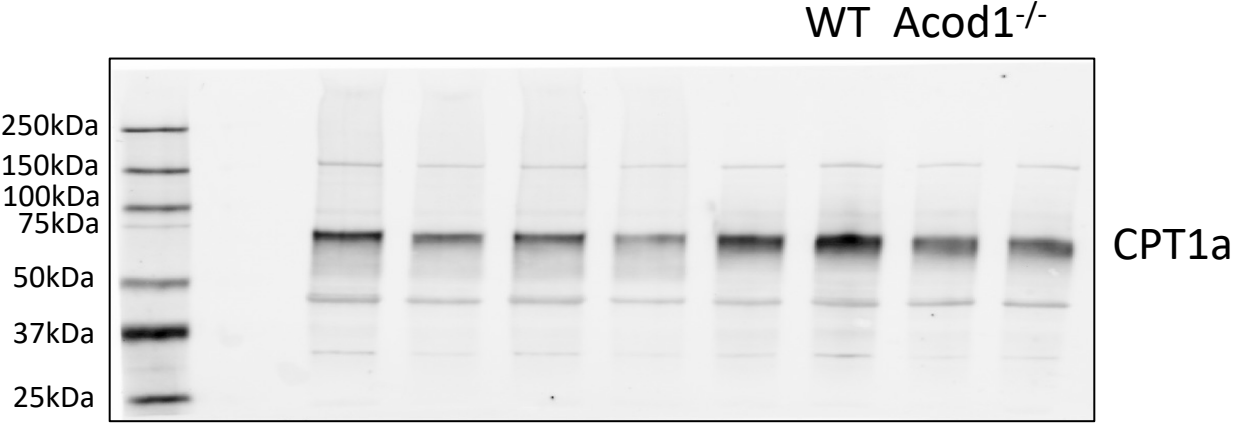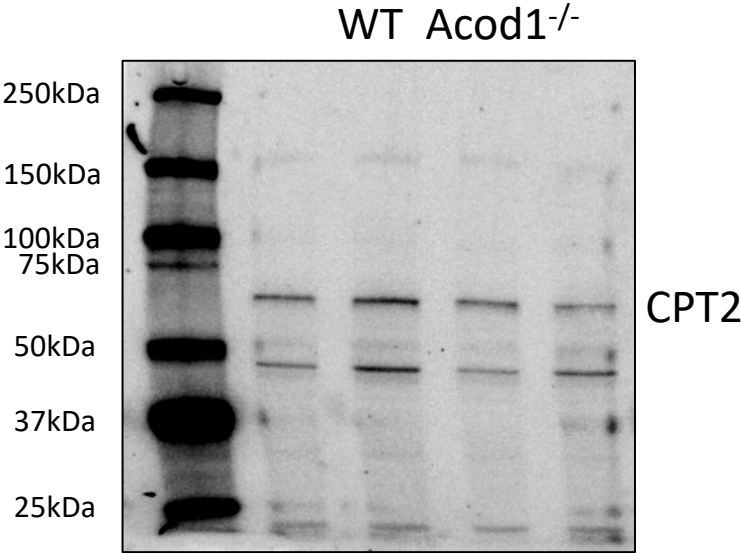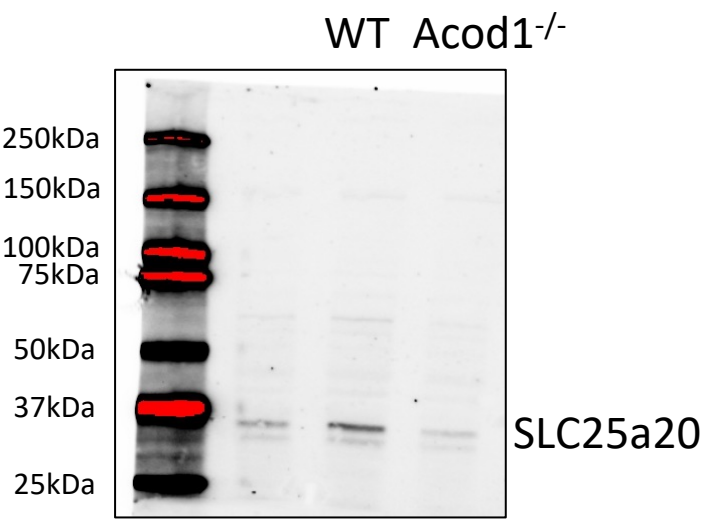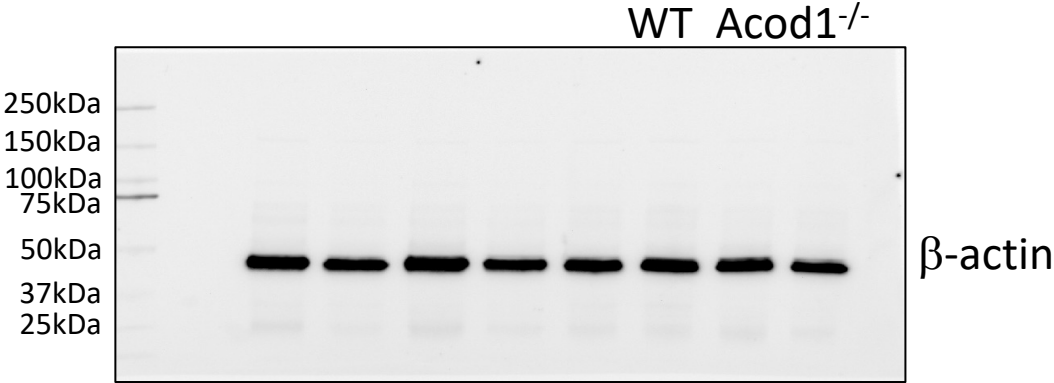

Supplement: Figure 2—source data 2. [file elife-92420-fig2-data2.pdf]

Figure 3a

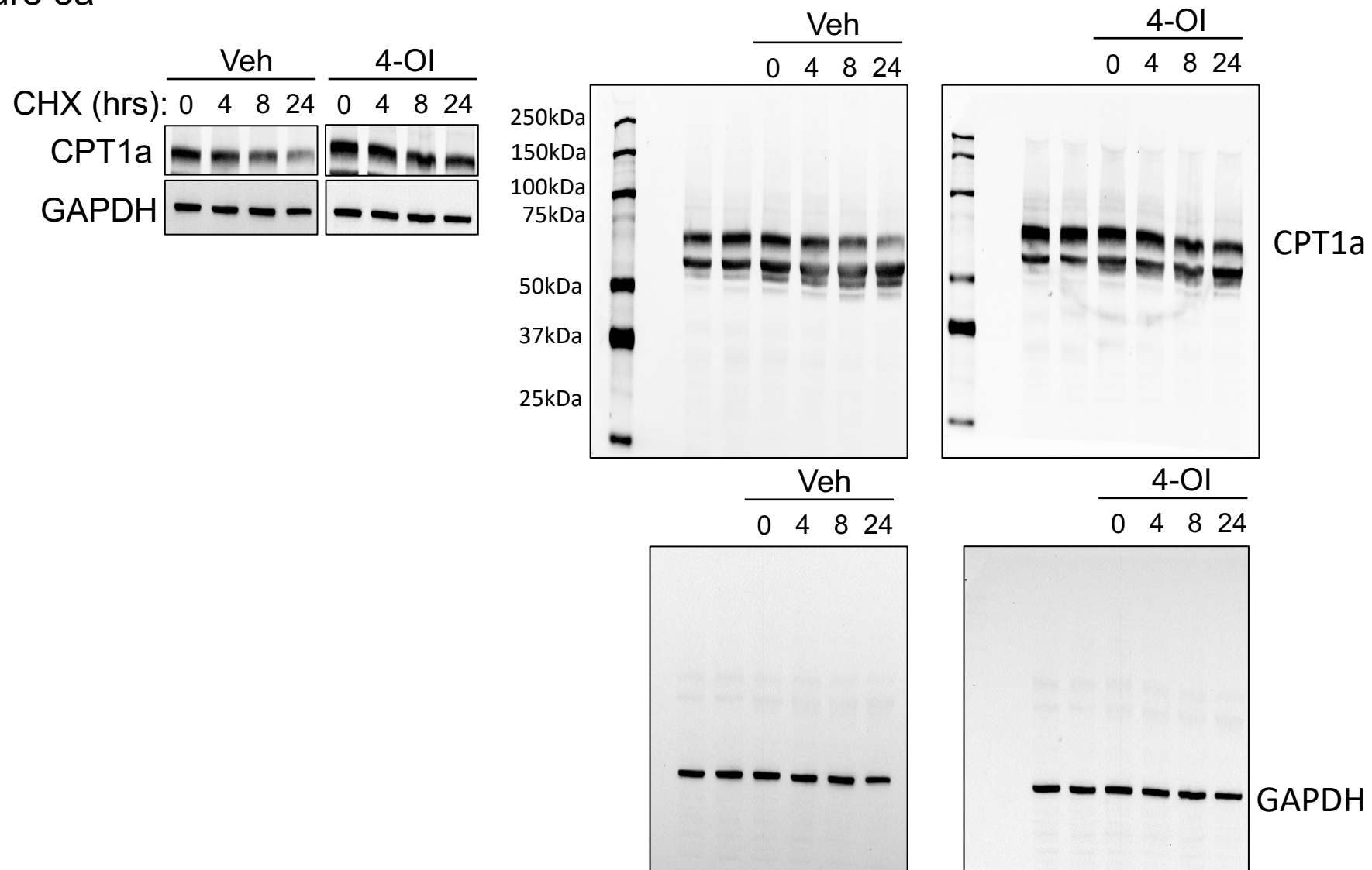

Supplement: Figure 3—source data 1. [file elife-92420-fig3-data1.pdf]

Figure 3b

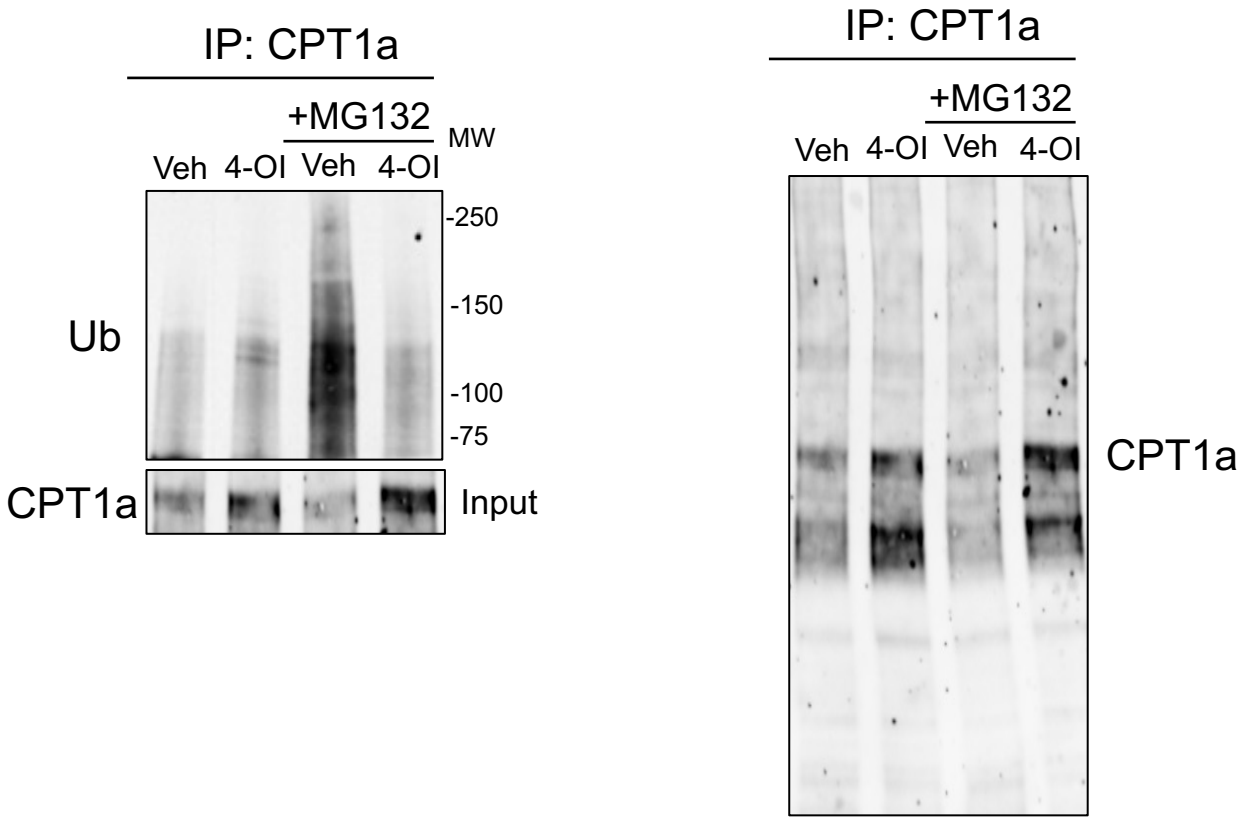

Supplement: Figure 3—source data 2. [file elife-92420-fig3-data2.pdf]

Figure 4b

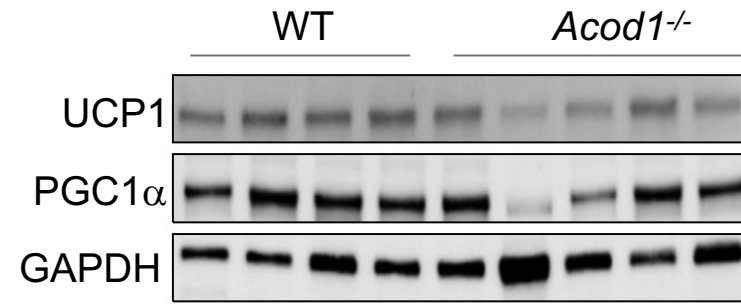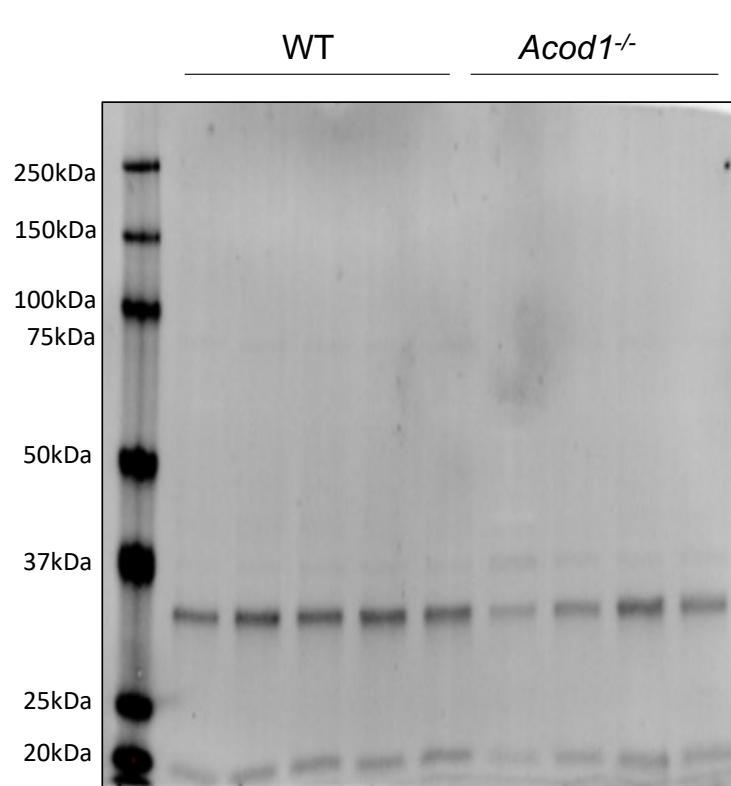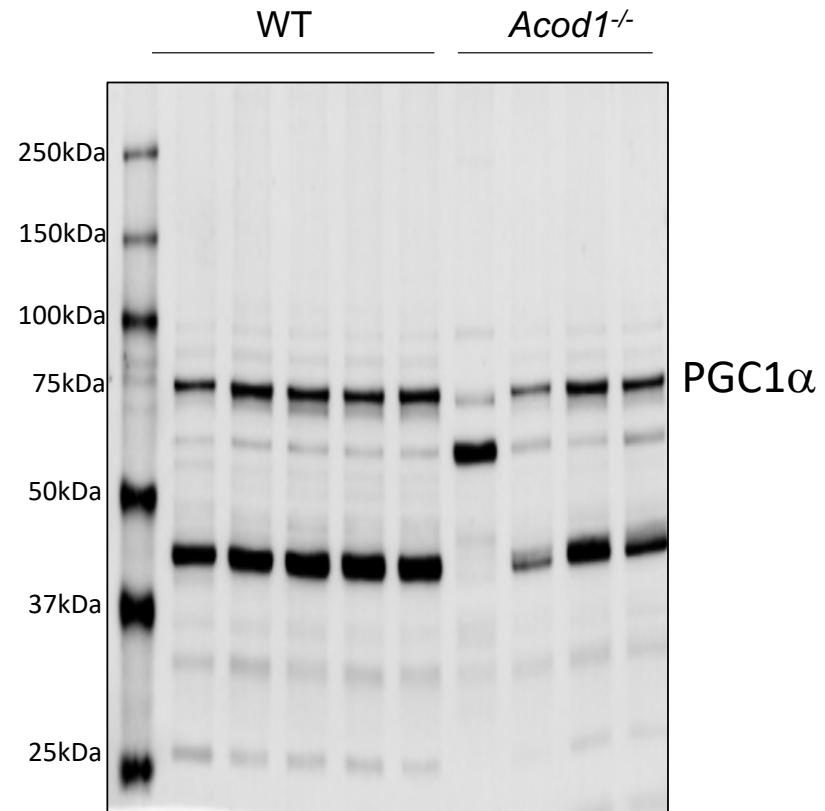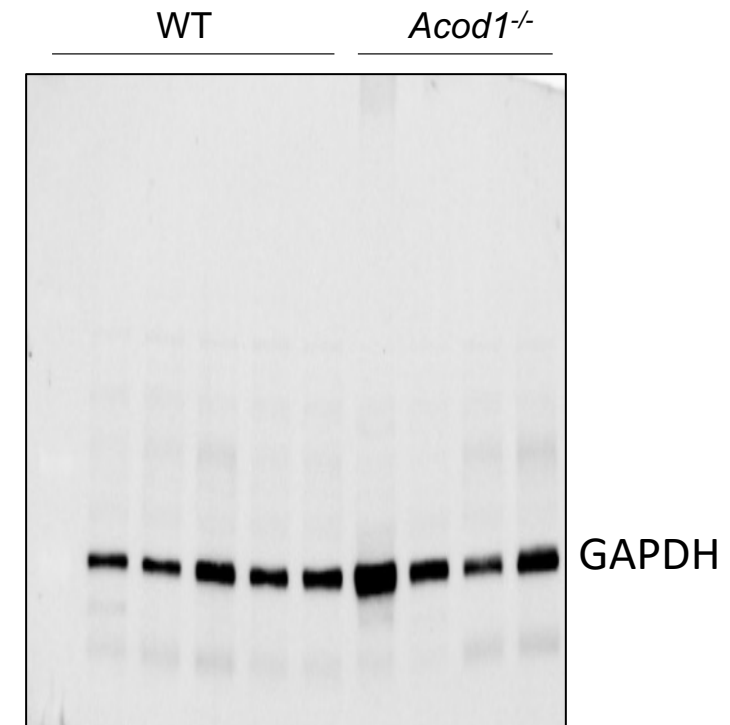

Supplement: Figure 4—source data 1. [file elife-92420-fig4-data1.pdf]
